# Club Foot.—III

**Published:** 1895-04-20

**Authors:** Walter L. Woolcombe

**Affiliations:** Assistant Surgeon South Devon and East Cornwall Hospital


### Medical Progress and Hospital Clinics.

[*The Editor will be glad to receive offers of co-operation and contributions from members of the profession. All letters should be addressed to* The Editor, The Lodge, Porchester Square, London, W.]

### CLUB FOOT.—III.

By Walter L. Woolcombe, F.R.C.S.E., Assistant Surgeon South Devon and East Cornwall Hospital.

(*Continued from page* 28).

The third part of my subject deals with the treatment of the deformities already described, and to this I originally intended to devote a considerable part of the paper, but I have taken so much time over the first part that I shall only indicate in the briefest manner the main principles at which we should aim.

The objects of our treatment are: (*a*) To remove the deformity; (*b*) to restore the function of the limb; and these principles apply alike to the congenital and acquired forms. In the former, the practitioner who delivers the child has an enormous advantage over anyone who sees it later, it may be not for several years, and incurs a corresponding responsibility, for during the first few months of life the body undergoes rapid development, and if the tarsal bones and muscles are allowed to develop in a faulty position, the difficulty of correcting it is enormously increased. (1) I would lay great stress on the fact that a great deal can be done at this early period, not by mechanical appliances, it is true, but by systematic, intelligent, and frequently-repeated movements by the nurse or mother under the guidance of the medical man, the movements being directed to stretching the contracted tendons and ligaments. In cases of congenital varus, although some more forcible means of correction is generally required later, yet a great deal of time can be saved, and a far more serviceable foot obtained eventually, if this treatment by manipulation is carried out in early infancy. (2) The next means in order of simplicity is bandaging to splints, and for these a great variety of material has been suggested. A very useful form for young children is malleable iron, which at the start is adjusted to the deformity and gradually straightened as the structures yield. (3) Plaster of Paris, which may be used in several ways, either applied after a moderate stretching and re-applied every three or four days, or else applied after forcible wrenching to the normal position. In this case it must be retained for from four to six weeks if the condition of the foot allows it. (4) Scarpa's shoe, or the modifications suggested by Adams, Little, Chance, &c. The principle of this is that a metal shoe is attached to the foot in the deformed position, and an iron upright to the leg, and then by means of a rack joint at the ankle the foot is brought gradually straight. This, if properly fitted, is a very useful adjunct to other treatment, but the objection is its expense. (5) Some arrangement of elastic bands, either fixed to the foot and leg by adhesive plaster, and made to act in the direction of the stretched muscles, or else attached to a moveable shoe and leg piece. These are useful in cases of infantile paralysis where the muscles have undergone fatty degeneration and cannot recover, otherwise the objection to their use is that the muscles are deprived of any stimulus to resume their function. (6) Tenotomy—the division of contracted tendons, fasciæ, ligaments, &c. The introduction of this means of treatment, or rather the making it a safe and easy procedure by doing it subcutaneously, has caused a great advance in the treatment of talipes and rendered capable of cure cases which were otherwise hopeless. It was first suggested by a physician—one Thilemus—in 1824, and he being a physician called in a surgeon named Lorenz to perform it on the tendo Achillis. It was done by an open wound and suppurated, but great improvement followed. Soon afterwards Sartorius repeated it and it is now our principal means of treatment. In discussing it I should like to urge the importance of a thorough division of all resisting tendons (in those cases where tenotomy is a necessity), and a prolonged and careful after-treatment; for we must not suppose that tenotomy alone will be sufficient; the second object of treatment, that of restoring the functions of the limb, has still to be attained. Those who have had to deal with relapsed cases will agree with me that the difficulty of a second tenotomy is incomparably greater than a primary one and much less satisfactory.

One reason why so many cases used to relapse is accounted for by the fact that before the mode of union of tendons was thoroughly demonstrated by Savory and others, it was considered dangerous to separate the divided ends to more than a very slight extent until time had been given for the formation of fibrous tissue between the cut surfaces, and thus most valuable time was lost. Personally, I do not consider that there is any danger of separating the ends too much in club-foot, except in the case of tendo Achillis. This should be put up at a right angle for a week and then gradually stretched. It will be found necessary to divide the performance of tenotomy into several stages in some forms of talipes, *e.g.*, T. varus. Here it is best to divide the tibials first, then after a week the plantar fascia, keeping during the performance of these two the tendo Achillis as a fulcrum. In yet another week the tendo Achillis should be divided. In dividing the contracted plantar fascia multiple punctures and division from the skin downwards is the best plan of procedure. The last means of correcting deformity at our disposal is the removal of bones or a portion of bone.

The best known operation of this class is that devised by Mr. Davy for T. varus, and consists in removing a wedge-shaped piece from the tarsus irrespective of what bones are divided, the amount to be taken being ascertained by cutting up plaster casts of the deformed feet. For the purpose of this operation Mr. Davy devised a set of wedges made of a polished steel frame grooved on the under surface to receive the end of a probe-pointed saw. These, after the free use of an elevator, were passed, from an incision on the outer side of the foot, between the tendons and bone across the front of the tarsus, and made to project through a puncture on the inner side, and the saw being passed first along one groove and then along the other, was made to cut through the tarsus on to a metal retractor passed beneath the tarsus. I have seen some excellent results from this, and have done two cases myself, but the cases must be very carefully selected, and there is a risk of being tempted to do it in cases that with patience can be cured without being exposed to the risks of such a severe operation. Mr. Davy now advises the use of a chisel through a wound on the outer side, and says that he frequently finds removal of a wedge from the cuboid, or the whole cuboid, sufficient. I cannot help thinking these cases might be quite well cured without the operation; personally, I have always experienced the greatest difficulty with the parts around the internal malleolus, which are not affected by this operation. I have only tried chiselling in one case, which I still have under treatment, and in this case, after removing most of the cuboid, I had to proceed, first to divide the neck of the astragalus, then to remove its head, and finally to chisel out a portion of the scaphoid, and even then the result was not very good in one of the feet. Removal of bone for other forms of talipes is not advisable; in T. equinus, the only other form in which it has been advised, so far as I am aware, the amount of bone that has to be removed is so great that it is not justifiable.

Finally, the treatment necessary to restore the functions of the limb consists (after the important stage of correcting the deformity, and thus putting the muscles in a position to act with advantage) in a judicious course of massage, electricity, and an apparatus which will prevent a recurrence of the displacement, and at the same time allow the muscles to act. I will not describe the various appliances which have been devised for this purpose, the best of which are associated with the names of Adams, Scarpa, Little, Tamplin, and Chance.

